# Prevalence of reflux-related symptoms in South-Hungarian blood donor volunteers

**DOI:** 10.1371/journal.pone.0265152

**Published:** 2022-03-15

**Authors:** Krisztina Helle, Lenke Bálint, Veronika Szekeres, Georgina Ollé, András Rosztóczy

**Affiliations:** 1 Division of Gastroenterology, First Department of Internal Medicine, University of Szeged, Szeged, Hungary; 2 Hungarian National Blood Transfusion Service, Szeged, Hungary; Sapienza University of Rome, ITALY

## Abstract

**Background/Aim:**

Population-based studies on the prevalence of GERD-related symptoms are still missing in Eastern Europe, therefore, we aimed to obtain such data in South-East Hungarian subjects.

**Methods:**

A total of 2,002 apparently healthy blood donor volunteers were consecutively enrolled and completed detailed questionnaires related to general factors, demographic data, socioeconomical factors, and the presence and frequency of typical and atypical GERD-related symptoms.

**Results:**

Among 2,002 study participants, 56.5% were completely asymptomatic. The prevalence of typical GERD symptoms appearing at least monthly or weekly was 16.5% and 6.8%, respectively. Two-thirds (209/330) of the patients experienced at least monthly occurring typical GERD symptoms and also had associated atypical symptoms and this was even more pronounced when comparing subgroups with higher symptom frequencies. Significant correlations were found between monthly GERD-related complaints and height, body mass index (BMI), coffee consumption, and smoking. Positive family history was another significant factor in all the symptom-frequency categories. GERD-related symptom frequency showed a linear association with sex (R^2^ = 0.75, P = 0.0049). Typical and atypical GERD symptoms were significantly more common in those with chronic diseases than those without. Heartburn was observed in 12.5% and 4.4% (*P*<0.05) and acid regurgitation was seen in 6.9% and 1.8% (*P*<0.05), respectively.

**Conclusion:**

The prevalence of GERD-related symptoms in South Hungary was significantly lower than that in Western countries and was closer to Eastern values. The presence of mild, non-exclusionary chronic diseases significantly increased the prevalence of GERD-related symptoms, as well as positive family history of GERD, height, BMI, coffee consumption, and smoking.

## Introduction

Gastroesophageal reflux disease (GERD) is one of the most common gastrointestinal diseases worldwide. It is a chronic condition in which frequent regurgitation of the gastric acid into the esophagus, mouth, and/or respiratory system causes typical (heartburn, regurgitation) and atypical (chronic cough, other respiratory symptoms, chest pain, dysphagia, globus sensation, nausea, vomiting) symptoms and/or esophageal/extraesophageal complications. These symptoms are common in the general population and have an impact on quality of life; however, only a few people consult a doctor about them [1–4].

The prevalence of GERD has been determined according to the presence of typical GERD symptoms in many epidemiological studies, although symptom-oriented diagnosis of GERD has at least two issues. First, these “typical symptoms” are also present in patients with functional esophageal disorders (e.g., functional heartburn), which cannot be subjectively distinguished from those caused by acidic reflux. This is supported by the recommendation of the Rome Foundation, as the presence of symptoms alone is not sufficient to diagnose functional esophageal disorders, and a more detailed evaluation is required [5, 6]. Second, many patients have not typical symptoms of GERD (mostly in Barrett’s esophagus or asthma), therefore a symptom-oriented diagnosis cannot be carried out at all, and detailed clinical evaluation is needed [7–13].

With the exception of these limitations, large epidemiological studies have shown that the prevalence of symptomatic GERD is around 20% to 25% in the Western world and 10% in Eastern countries [14–16]. Little, if anything, is known about the prevalence in central Europe, which is located between the west and east, and is where a substantial part of the population lives outside of the larger cities.

The present study aimed to obtain population-based data on the prevalence of GERD-related symptoms in South-East Hungary to identify potential risk factors for the presence of GERD-related symptoms and compare the data with the known Western and Eastern values.

## Patients and methods

A total of 2,002 apparently healthy, health-conscious, unremunerated blood donor volunteers [1,156 (42.1) males and 846 (57.9%) females; mean age, 39 (18–65) years] were consecutively enrolled, after given informed consent in written form. Data were collected by means of a questionnaire at the Hungarian National Blood Transfusion Service in Szeged and in the settlements of Csongrád-Csanád county. In Hungary, blood donation from healthy people who weigh >50 kg and are aged between 18 and 65 years is permitted. Volunteers are allowed to have the following diseases in initial and/or mild/well-controlled form: hypertension (at target value with antihypertensive monotherapy), diabetes mellitus (normal serum glucose and HbA1c levels with diet ± metformin), obesity, hypothyroidism/hyperthyroidism (normal thyroid gland function with therapy), hypercholesterolemia (at normal value with diet ± statin), hyperuricemia, asthma bronchiale (normal respiratory function test with long and short-acting bronchodilator inhalers, and/or with other medication), allergy (intermittent antihistamine therapy), GERD, osteoporosis, tachycardia/arrhythmias, polycystic ovary syndrome, coeliac disease, eczema, Gilbert’s syndrome, and some musculoskeletal disorders [17]. These conditions were confirmed by the physician of the Hungarian National Blood Transfusion Service.

Participant donors completed detailed questionnaires related to general factors [age, sex, body mass index (BMI) calculated from height and weight], demographic data (place of childhood, current place of residence, composition of the family, occupation), socioeconomical factors (smoking habits, alcohol and coffee consumption, family history of GERD, patient history of chronic diseases). The presence and frequency of typical (heartburn, acid regurgitation) and atypical GERD-related symptoms (nausea, dysphagia, globus sensation, respiratory symptoms as chronic cough, shortness of breath, hoarseness, new or worsening asthma, and chest pain) were also assessed. The following symptom-frequency categories were used: at least once a day, at least once a week, at least once a month, and less than once a month.

Subgroup analysis was performed by age, sex, height, weight, BMI, smoking habits, alcohol and coffee consumption, inheritance (GERD in the family), chronic diseases, and prevalence of symptoms. All statistical analyses were performed using R software. Chi-square test, unpaired *t* test, and linear regression were applied as required. The level of significance was set at 0.05. The study received ethical approval (ethical committee approval number: WHO 3345).

## Results

**Fig 1:**
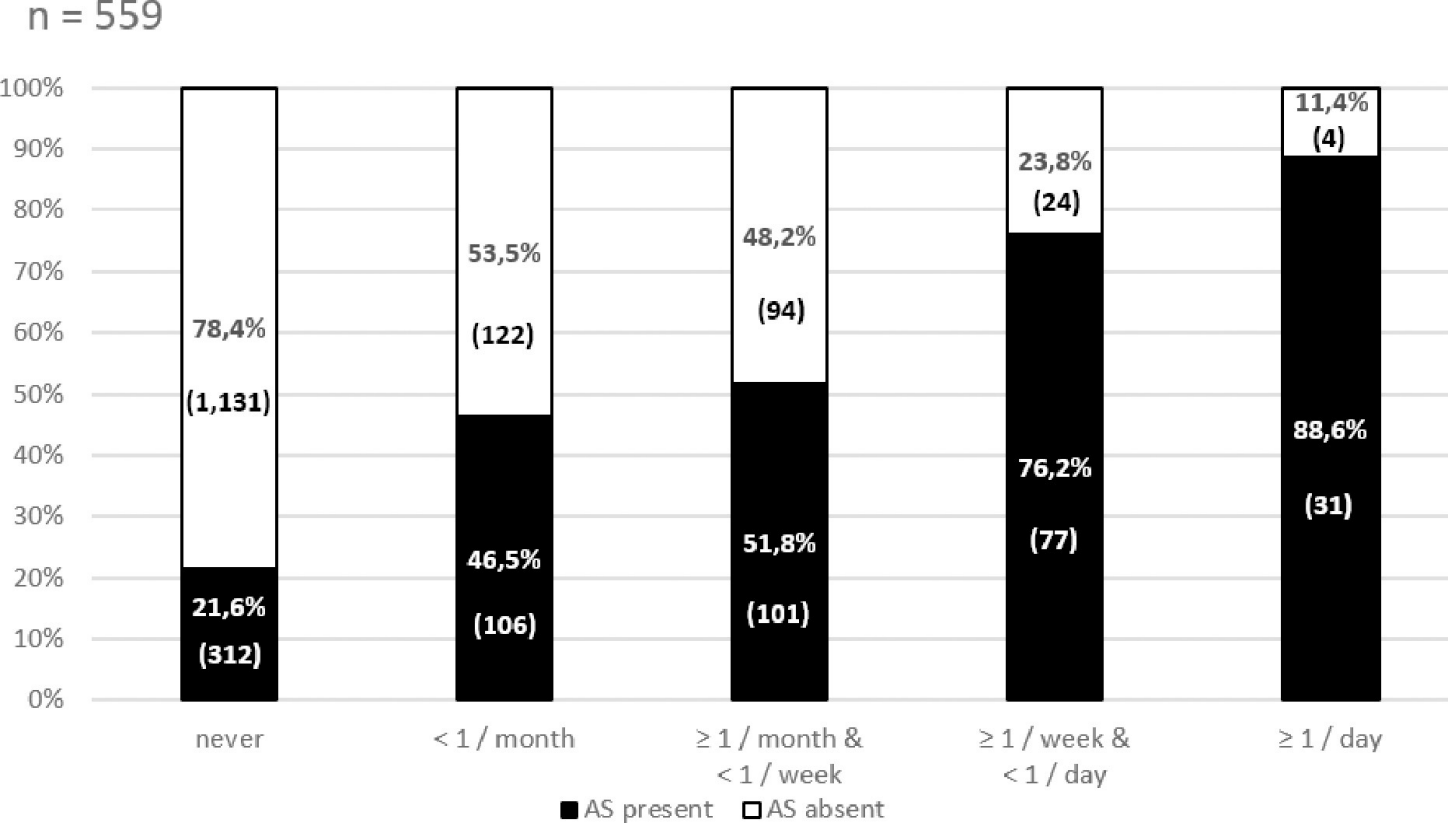
The occurrence of atypical symptoms in participants with typical GERD symptoms. There is a positive linear association between the frequency of the typical GERD-related symptom, and the presence of atypical symptoms (AS). (R^2^ = 0.9748, P<0.0001).

Among the 2,002 consecutive blood donor volunteers, 56.5% (1,131/2,002) were completely asymptomatic. Among the remaining volunteers, 27.9% (559/2,002) had typical symptoms of GERD (heartburn and/or acidic regurgitation). However, symptoms that appeared at least monthly or weekly were significantly less common [16.5% (330/2,002) and 6.8% (136/2,002), P < 0.05, respectively]. The majority of participants with typical GERD symptoms [56.4% (315/559)] also had atypical symptoms (such as abdominal pain, nausea, vomiting, dysphagia, globus sensation, cough, respiratory symptoms, and chest pain). This difference was further and significantly increased and showed a linear correlation with symptom frequency (R^2^ = 0.9748, P < 0.0001) (Fig 1). Atypical symptoms were also seen in participants that had typical GERD symptoms at least monthly [63.1% (209/330)], weekly [79.4% (108/136)], and daily [88.6% (31/35)] (Table 1).

**Table 1:** The presence of the typical and/or atypical symptoms.

n = 2,002		ever	< 1 / month	≥ 1 / month & < 1 / week	≥ 1 / week & < 1 / day	≥ 1 / day
typical GERD symptoms	atypical GERD symptoms					
***present (n = 559)***	***present***	315 *(15*.*7%)*	106 *(5*.*3%)*	101 *(5*.*0%)*	77 *(3*.*8%)*	31 *(1*.*6%)*
	***absent***	244 *(12*.*2%)*	122 *(6*.*1%)*	94 *(4*.*7%)*	24 *(1*.*2%)*	4 *(0*.*2%)*
***absent (n = 1*,*443)***	***present***	312 *(15*.*6%)*				
	***absent***	1,131 *(56*.*5%)*				

Detailed symptom analysis showed that the prevalence of heartburn was higher than that of acidic regurgitation in all symptom-frequency categories.

Among the atypical (esophageal and extraesophageal) symptoms, respiratory symptoms were the most prevalent (19%), although only 13% of the participants had respiratory symptoms at least monthly. Globus sensation occurred in 6% and other atypical symptoms were reported by <5% of the participants (Table 2).

**Table 2:** The prevalence and frequency of different GERD-related symptoms.

n = 2,002	< 1 / month	≥ 1 / month & < 1 / week	≥ 1 / week & < 1 / day	≥ 1 / day
***heartburn***	168 *(8*.*4%)*	160 *(8*.*0%)*	93 *(4*.*6%)*	29 *(1*.*4%)*
***acid regurgitation***	163 *(8*.*1%)*	108 *(5*.*4%)*	44 *(2*.*2%)*	18 *(0*.*9%)*
***typical symptoms during night***	51 *(2*.*5%)*	32 *(1*.*6%)*	20 *(1*.*0%)*	5 *(0*.*2%)*
***dysphagia***	8 *(0*.*4%)*	12 *(0*.*6%)*	13 *(0*.*6%)*	4 *(0*.*2%)*
***globus sensation***	54 *(2*.*7%)*	33 *(1*.*6%)*	23 *(1*.*1%)*	9 *(0*.*4%)*
***upper respiratory tract symptoms***	121 *(6*.*0%)*	80 *(4*.*0%)*	80 *(4*.*0%)*	93 *(4*.*6%)*
***dyspnea***	35 *(1*.*7%)*	14 *(0*.*7%)*	16 *(0*.*8%)*	3 *(0*.*1%)*
***chest pain***	26 *(1*.*3%)*	40 *(2*.*0%)*	11 *(0*.*5%)*	4 *(0*.*2%)*
***epigastric pain***	66 *(3*.*3%)*	56 *(2*.*8%)*	60 *(3*.*0%)*	21 *(1*.*0%)*
***abdominal pain***	8 *(0*.*4%)*	19 *(0*.*9%)*	24 *(1*.*2%)*	11 *(0*.*5%)*
***nausea*, *vomiting***	37 *(1*.*8%)*	27 *(1*.*3%)*	20 *(1*.*0%)*	4 *(0*.*2%)*

**Fig 2:**
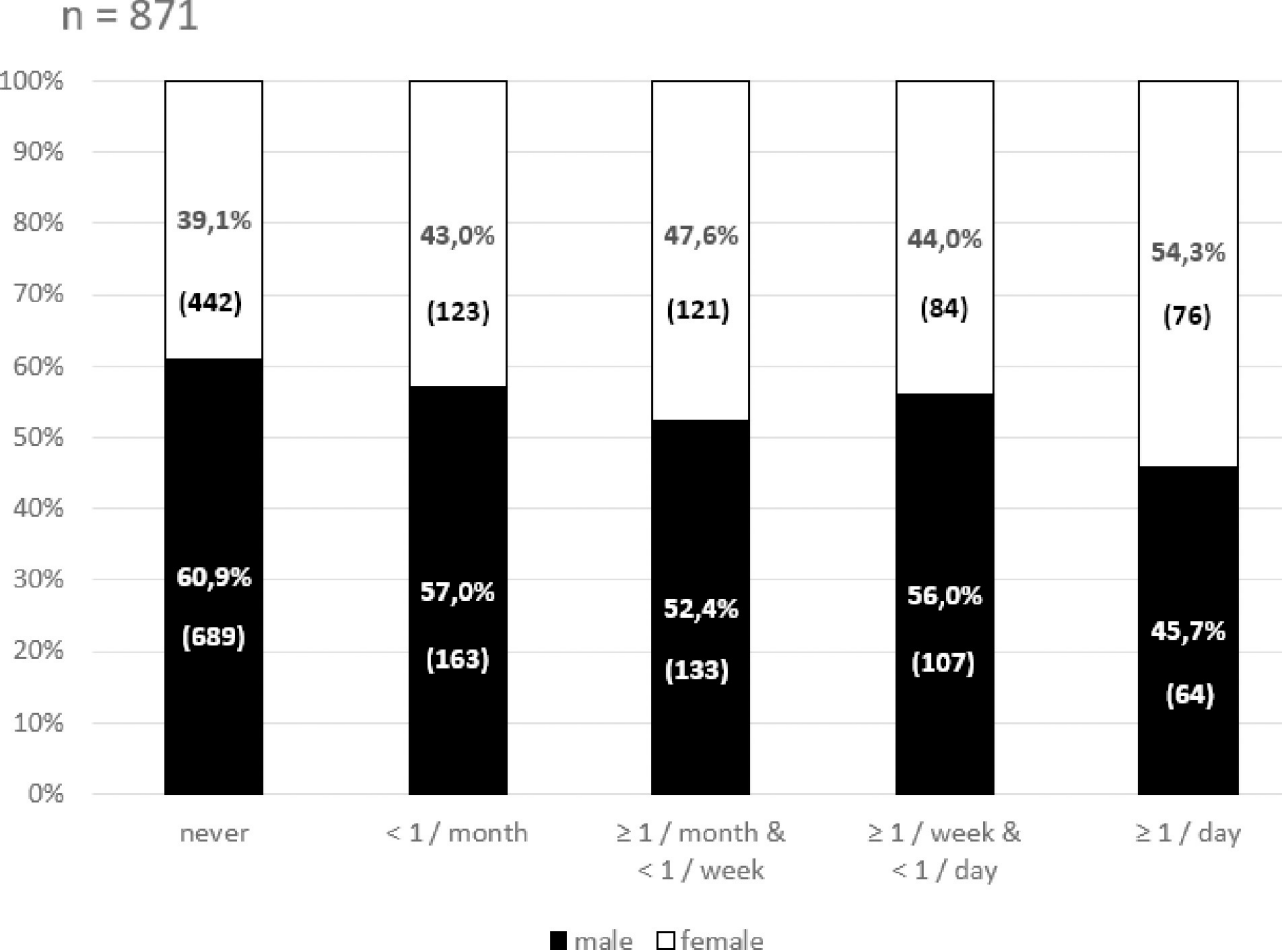
The frequency of GERD-related symptoms is positively associated to female sex (R^2^ = 0.75, P = 0.0049). Similar association was found if only the typical symptoms were assessed.

Among the blood donors who had GERD-related symptoms at least monthly, significant correlations were found between the complaints and some socioeconomical factors, such as height, BMI, coffee consumption, and smoking. The correlation between frequency of symptoms and sex of the volunteers was different. Females showed an increased frequency of GERD-related symptoms (R^2^ = 0.75, P = 0.0049) (Fig 2). This linear association was observed when both typical and all symptoms were assessed (Fig 2). The remaining parameters (occupation, household population, current, and childhood residence) showed no correlation. Positive family history was a significant predictive factor in all studied symptom-frequency categories (Table 3). Analysis of typical GERD-related symptoms only showed the tendency was the same as in blood donors with any GERD-related symptoms (Table 4).

**Table 3:** Socioeconomical factors of blood donor volunteers with any GERD-related symptoms (typical and/or atypical). Age, weight, height and BMI are presented with mean ± SD and range.

	Asymptomatic *(n = 1*,*131)*	Any GERD-related symptoms (typical / atypical) *(n = 871)*			
		< 1 / month *(n = 286)*	≥ 1 / month & < 1 / week *(n = 254)*	≥ 1 / week & < 1 / day *(n = 191)*	≥ 1 / day *(n = 140)*
**Sex**					
Male	689 (60.9%)	163 (57.0%)	133 (52.4%)	107 (56.0%)	64 (45.7%)
Female	442 (39.1%)	123 (43.0%)	121 (47.6%) *	84 (44.0%)	76 (54.3%) **
**Age (years)**	39 ± 12.4 (17–66)	39 ± 12.7 (17–65)	38 ± 13.3 (18–65)	38 ± 12.3 (18–64)	41 ± 12.2 (18–64)
**Weight (kg)**	81 ± 15.8 (50–140)	81 ± 16.7 (52–135)	80 ± 17.2 (51–180)	81 ± 17.0 (52–130)	79 ± 16.9 (50–140)
**Height (cm)**	174 ± 9.2 (150–197)	173 ± 8.9 (155–198)	172 ± 9.5 (150–197) **	173 ± 9.1 (150–196)	171 ± 9.9 (150–200) **
**BMI (kg/m** ^**2**^ **)**	26.6 ± 4.5 (16.9–52.7)	27.0 ± 4.8 (18.9–45.2)	27.0 ± 5.2 (17.7–52.6)	27.0 ± 4.8 (18–43.9)	26.9 ± 4.9 (17.0–45.2)
**BMI categories**					
1 –underweight	3 (0.3%)	0 (0.0%)	3 (1.0%)	2 (1.0%)	1 (0.7%)
2 –normal weight	444 (39.3%)	112 (39.2%)	96 (37.8%)	71 (37.2%)	57 (40.7%)
3 –overweight	455 (40.2%)	109 (38.1%)	99 (39.0%)	73 (38.2%)	43 (30.7%)
4 –obesity	169 (14.9%)	45 (15.7%)	39 (15.4%)	36 (18.8%)	32 (22.9%)
5 –extreme obesity	60 (5.3%)	20 (7.0%)	17 (6.7%)	9 (4.7%)	7 (5.0%) *
**Smoking**					
recent / previous	368 (32.5%)	102 (35.7%)	106 (41.7%)	84 (44.0%)	72 (51.4%)
never	763 (67.5%)	184 (64.3%)	148 (58.3%) **	107 (56.0%) **	68 (48.6%) **
**Coffee**					
yes	756 (66.8%)	195 (68.2%)	187 (73.6%)	132 (69.1%)	110 (78.6%)
no	375 (33.2%)	91 (31.8%)	67 (26.4%) *	59 (30.9%)	30 (21.4%) **
**Alcohol**					
regular	30 (2.7%)	8 (2.8%)	10 (3.9%)	9 (4.7%)	9 (6.4%)
never / occasional	1101 (97.3%)	278 (97.2%)	244 (96.1%)	182 (95.3%)	131 (93.6%)
**GERD in the family**					
yes	147 (13.0%)	54 (18.9%) **	63 (24.8%) **	54 (28.3%) **	39 (27.9%) **
no	825 (72.9%)	176 (61.5%)	154 (60.6%)	110 (57.6%)	77 (55.0%)
unknown	159 (14.1%)	56 (19.6%)	37 (14.6%)	27 (14.1%)	24 (17.1%)

*: p < 0.05, compared to the asymptomatic subjects

**: p < 0.01, compared to the asymptomatic subjects

**Table 4:** Socioeconomical factors of blood donor volunteers with any GERD-related typical symptoms. Age, weight, height and BMI are presented with mean ± SD and range.

	Asymptomatic *(n = 1*,*131)*	GERD-related typical symptoms *(n = 559)*			
		< 1 / month *(n = 229)*	≥ 1 / month & < 1 / week *(n = 194)*	≥ 1 / week & < 1 / day *(n = 101)*	≥ 1 / day *(n = 35)*
**Sex**					
Male	689 (60.9%)	145 (63.3%)	100 (51.5%)	54 (53.5%)	16 (45.7%)
Female	442 (39.1%)	84 (36.7%)	94 (48.5%) **	47 (46.5%)	19 (54.3%)
**Age (years)**	39 ± 12.4 (17–66)	39 ± 12.5 (18–65)	39 ± 12.9 (18–64)	41 ± 11.6 (18–64)	39 ± 10.9 (21–58)
**Weight (kg)**	81 ± 15.8 (50–140)	83 ± 16.7 (51–135)	83 ± 18.3 (51–180)	82 ± 15.7 (54–130)	82 ± 20.9 (50–140)
**Height (cm)**	174 ± 9.2 (150–197)	174 ± 9.4 (153–198)	172 ± 9.7 (150–200) **	172 ± 8.7 (150–190) *	172 ± 9.0 (153–187)
**BMI (kg/m** ^**2**^ **)**	26.6 ± 4.5 (16.9–52.7)	27.2 ± 5.0 (17.7–45.2)	27.9 ± 5.4 (18.3–52.6) **	27.7 ± 4.5 (18.8–39.7) *	27.6 ± 5.7 (20.1–45.2)
**BMI categories**					
1 –underweight	3 (0.3%)	3 (1.3%)	1 (0.5%)	0 (0%)	0 (0%)
2 –normal weight	444 (39.3%)	84 (36.7%)	66 (34.0%)	33 (32.7%)	11 (31.4%)
3 –overweight	455 (40.2%)	84 (36.7%)	73 (37.6%)	39 (38.6%)	14 (40.0%)
4 –obesity	169 (14.9%)	40 (17.5%)	36 (18.6%)	26 (25.7%)	7 (20.0%)
5 –extreme obesity	60 (5.3%)	18 (7.9%) *	18 (9.3%)	3 (3.0%)	3 (8.6%)
**Smoking**					
recently/previously	368 (32.5%)	80 (34.9%)	77 (39.7%)	44 (43.6%)	14 (40.0%)
never	763 (67.5%)	149 (65.1%)	117 (60.3%) *	57 (56.4%) *	21 (60.0%)
**Coffee**					
yes	756 (66.8%)	151 (65.9%)	145 (74.7%)	77 (76.2%)	25 (71.4%)
no	375 (33.2%)	78 (34.1%)	49 (25.3%) *	24 (23.8%) *	10 (28.6%)
**Alcohol**					
regularly	30 (2.7%)	6 (2.6%)	9 (4.6%)	6 (5.9%)	2 (5.7%)
never/occasionally	1101 (97.3%)	223 (97.4%)	185 (95.4%)	95 (94.1%)	33 (94.3%)
**GERD in the family**					
yes	147 (13.0%)	52 (22.7%)	49 (25.3%)	43 (42.6%)	13 (37.1%)
no	825 (72.9%)	133 (58.1%)	107 (55.2%)	47 (46.5%)	17 (48.6%)
unknown	159 (14.1%)	44 (19.2%) **	38 (19.6%) **	11 (10.9%) **	5 (14.3%) **

*: p < 0.05, compared to the asymptomatic subjects

**: p < 0.01, compared to the asymptomatic subjects

Associations between different chronic diseases and GERD-related symptoms were examined. Due to the rules of eligibility to donate blood, volunteers with mild, well-controlled, chronic diseases were not excluded. Therefore, 390 participants with non-exclusionary diseases were also enrolled. Among these, 93.3% (364/390) had only one disorder, whereas 6.7% (26/390) had two different chronic conditions.

Among blood donors with non-exclusionary chronic diseases (e.g., hypertension, hyperthyroidism/hypothyroidism, diabetes), those with typical GERD symptoms appearing at least weekly were significantly more common compared with those without non-exclusionary chronic diseases. Heartburn and acid regurgitation were reported by 12.5% and 6.9% of participants with chronic diseases, in contrast to 4.4% and 1.8% of completely healthy participants, respectively (for all categories *P* < 0.05).

Overweight or obesity without any further chronic diseases were detected in 925/2,002 (46.2%) participants and showed no relevant effects on the prevalence of GERD-related typical symptoms (Table 5).

**Table 5:** The effect of coexisting, chronic diseases (including obesity) on the prevalence of GERD-related symptoms.

	with chronic diseases *(n = 390)*				without chronic diseases							
					BMI < 25 kg/m^2^ *(n = 687)*				BMI > 25 kg/m^2^ *(n = 925)*			
	< 1 / month	≥ 1 / month & < 1 / week	≥ 1 / week & < 1 / day	≥ 1 / day	< 1 / month	≥ 1 / month & < 1 / week	≥ 1 / week & < 1 /day	≥ 1 /day	< 1 /month	≥ 1 / month & < 1 /week	≥ 1 /week & < 1 /day	≥ 1 /day
**heartburn**	39 *(10*.*0%)*	33 *(8*.*5%)*	38 *(9*.*7%)* **	11 *(2*.*8%)* *	47 *(6*.*8%)*	47 *(6*.*8%)*	22 *(3*.*2%)*	8 *(1*.*2%)*	82 *(8*.*9%)*	80 *(8*.*6%)*	33 *(3*.*6%)*	10 *(1*.*1%)*
**acid regurgitation**	42 *(10*.*8%)* *	34 *(8*.*7%)* **	16 *(4*.*1%)* *	11 *(2*.*8%)* **	40 *(5*.*8%)*	28 *(4*.*1%)*	12 *(1*.*7%)*	1 *(0*.*1%)*	81 *(8*.*8%)*	46 *(5*.*0%)*	16 *(1*.*7%)*	6 *(0*.*6%)*
**typical symptoms during night**	18 *(4*.*6%)* **	8 *(2*.*1%)*	8 *(2*.*1%)* *	3 *(0*.*8%)*	5 *(0*.*7%)*	9 *(1*.*3%)*	4 *(0*.*6%)*	1 *(0*.*1%)*	28 *(3*.*0%)*	15 *(1*.*6%)*	8 *(0*.*9%)*	1 *(0*.*1%)*
**dysphagia**	4 *(1*.*0%)*	5 *(1*.*3%)*	7 *(1*.*8%)* *	2 *(0*.*5%)*	1 *(0*.*1%)*	4 *(0*.*6%)*	3 *(0*.*4%)*	1 *(0*.*1%)*	3 *(0*.*3%)*	3 *(0*.*3%)*	3 *(0*.*3%)*	1 *(0*.*1%)*
**globus sensation**	16 *(4*.*1%)* *	9 *(2*.*3%)*	7 *(1*.*8%)*	6 *(1*.*5%)* **	21 *(3*.*1%)*	15 *(2*.*2%)*	7 *(1*.*0%)*	3 *(0*.*4%)*	17 *(1*.*8%)*	9 *(1*.*0%)*	9 *(1*.*0%)*	0 *(0%)*
**upper respiratory tract symptoms**	28 *(7*.*2%)*	20 *(5*.*1%)*	20 *(5*.*1%)*	33 *(8*.*5%)* **	46 *(6*.*7%)*	24 *(3*.*5%)*	30 *(4*.*4%)*	23 *(3*.*3%)*	47 *(5*.*1%)*	36 *(3*.*9%)*	30 *(3*.*2%)*	37 *(4*.*0%)*
**dyspnea**	17 *(4*.*4%)* **	8 *(2*.*1%)* **	7 *(1*.*8%)* *	3 *(0*.*8%)* **	5 *(0*.*7%)*	2 *(0*.*3%)*	5 *(0*.*7%)*	0 *(0%)*	13 *(1*.*4%)*	4 *(0*.*4%)*	4 *(0*.*4%)*	0 *(0%)*
**chest pain**	11 *(2*.8%)*	14 *(3*.*6%)* *	3 *(0*.*8%)* *	3 *(0*.*8%)* *	7 *(1*.*0%)*	13 *(1*.*9%)*	7 *(1*.*0%)* *	1 *(0*.*1%)*	8 *(0*.*9%)*	13 *(1*.*4%)*	1 *(0*.*1%)*	0 *(0%)*
**epigastric pain**	21 *(5*.4%)*	11 *(2*.*8%)*	25 *(6*.*4%)* **	11 *(2*.*8%)* **	16 *(2*.*3%)*	22 *(3*.*2%)*	14 *(2*.*0%)*	3 *(0*.*4%)*	29 *(3*.*1%)*	23 *(2*.*5%)*	21 *(2*.*3%)*	7 *(0*.*8%)*
**abdominal pain**	2 *(0*.*5%)*	5 *(1*.*3%)*	10 *(2*.*6%)* **	6 *(1*.*5%)* *	5 *(0*.*7%)*	7 *(1*.*0%)*	9 *(1*.*3%)*	2 *(0*.*3%)*	1 *(0*.*1%)*	7 *(0*.*8%)*	5 *(0*.*5%)*	3 *(0*.*3%)*
**nausea & vomiting**	12 *(3*.*1%)*	8 *(2*.*1%)*	10 *(2*.*6%)* **	2 *(0*.*5%)*	11 *(1*.*6%)*	12 *(1*.*7%)*	4 *(0*.*6%)*	2 *(0*.*3%)*	14 *(1*.*5%)*	7 *(0*.*8%)*	6 *(0*.*6%)*	0 *(0%)*

*: p < 0.05, compared to the respective group of subjects without chronic diseases

**: p < 0.01, compared to the respective group of subjects without chronic diseases.

**Fig 3:**
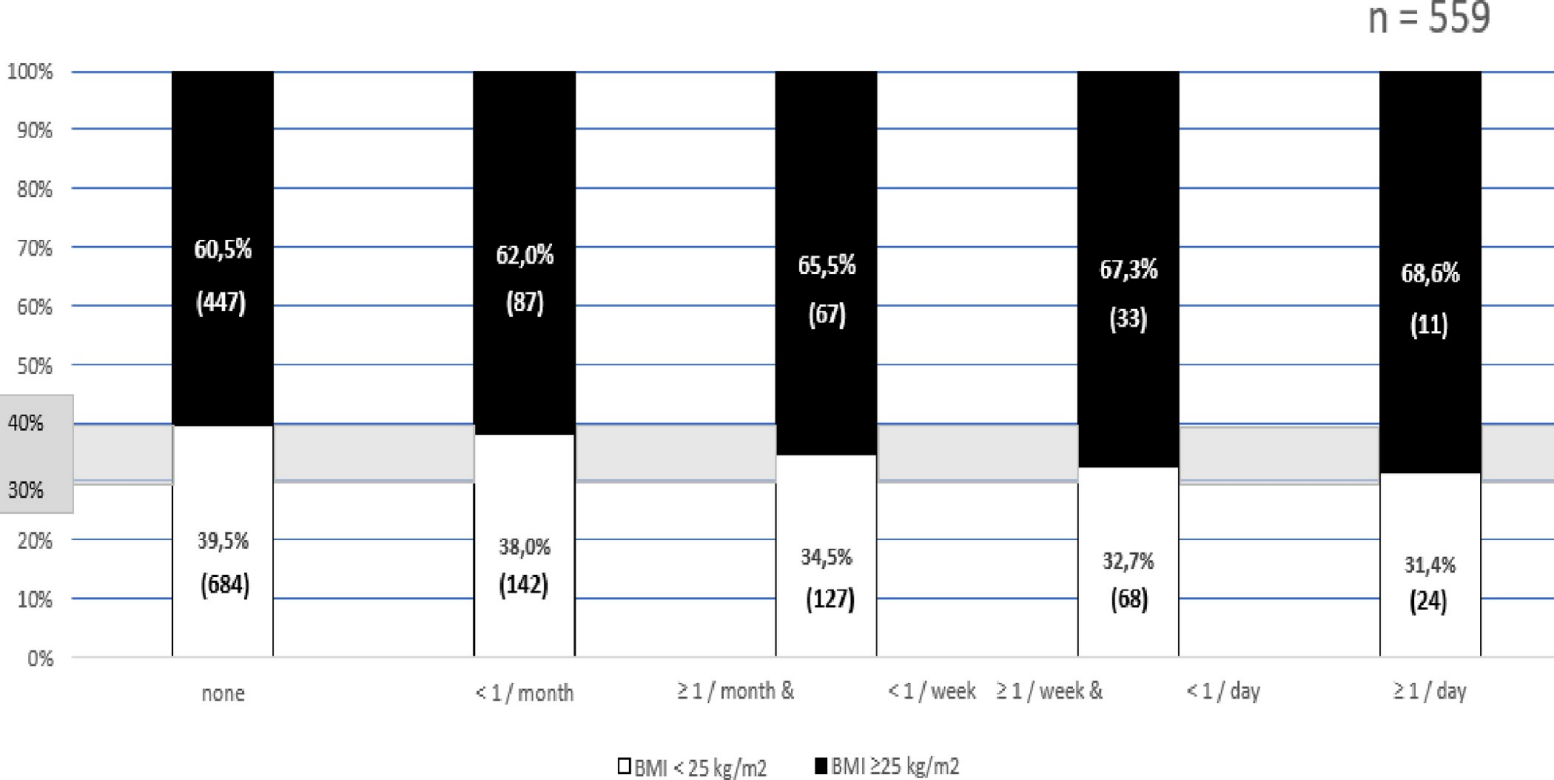
The frequency of GERD-related typical symptoms is positively associated to the presence of overweight (R^2^ = 0.63, P = 0.0497). Similar association was found if all (typical + atypical) reflux related symptoms were assessed.

On the contrary, blood donors with any GERD-related symptoms were more obese than asymptomatic participants and a linear correlation was observed between these two parameters (R^2^ = 0.63, P = 0.0497) (Tables 3 and 4, Fig 3).

In participants with hypertension (173/2,002, 8.6%), heartburn (45/173, 26%, *P* = 0.0313), acidic regurgitation (35/173, 20.2%, *P* = 0.0055), nocturnal typical symptoms (14/173, 8.1%, *P* = 0.009), and respiratory symptoms (42/173, 24.3%, *P* = 0.0385) were the most common complaints and were also significantly more prevalent compared with participants without chronic non-exclusionary disorders.

All typical and atypical symptoms were significantly more common in participants with known minor respiratory diseases [44/2,002 (2.2%); *P* < 0.05]. The low number of cases did not allow for statistical confirmation of the association with a positive family history of GERD (10/44, 22.7% vs. 27/44, 61.4%).

Globus sensation was the only considerable symptom (4/23, 17.4% *vs* 5.7%, *P* = 0.0434) among participants with thyroid disorders (23/2,002, 1.2%). Any type of cardiac disease (58/2,002, 2.9%) was associated with nausea, vomiting, and chest pain (*P* < 0.05).

Patients with non-exclusionary chronic diseases were older than completely healthy participants (Table 6). However, age showed no correlation with prevalence of GERD-related symptoms (Table 2).

**Table 6:** Socioeconomical factors of blood donor volunteers according to the presence of coexisting, chronic diseases. Age, weight, height, and BMI are presented with mean ± SD and range.

	with chronic diseases *(n = 390)*	without chronic diseases	
		BMI < 25 kg/m^2^ *(n = 687)*	BMI > 25 kg/m^2^ *(n = 925)*
**Sex**			
Male	205 (52.6%)	333 (48.5%)	618 (66.8%) **
Female	185 (47.4%)	354 (51.5%)	307 (33.2%)
**Age (years)**	45 ± 11.8 (18–66) **	34 ± 12.0 (18–65)	40 ± 12.0 (17–65)
**Weight (kg)**	85 ± 17.0 (52–134)	68 ± 9.1 (50–92)	89 ± 14.0 (58–180)
**Height (cm)**	172 ± 9.4 (150–198)	173 ± 8.9 (153–197)	174 ± 9.3 (150–200)
**BMI (kg/m** ^**2**^ **)**	28.7 ± 4.9 (17.0–45.2)	22.5 ± 1.7 (16.9–24.9)	29.2 ± 3.8 (25.0–52.7)
**BMI categories**			
1 –underweight	2 (0.5%)	7 (1.0%)	0
2 –normal weight	100 (25.6%)	680 (99.0%)	0
3 –overweight	141 (36.2%)	0	638 (69.0%)
4 –obesity	110 (28.2%)	0	211 (22.8%)
5 –extreme obesity	37 (9.5%)	0	76 (8.2%)
**Smoking**			
recently** **/** **previously	139 (35.6%)	248 (36.1%)	345 (37.3%)
never	251 (64.4%)	439 (63.9%)	579 (62.6%)
**Coffee**			
yes	285 (73.1%) *	457 (66.5%)	638 (69.1%)
no	105 (26.9%)	230 (33.5%)	286 (30.9%)
**Alcohol**			
regularly	15 (3.8%)	16 (2.3%)	35 (3.8%)
never** **/** **occasionally	375 (96.2%)	671 (97.7%)	889 (96.2%)
**GERD in the family**			
yes	87 (22.3%) *	119 (17.3%)	151 (16.3%)
no	244 (62.6%)	475 (69.1%)	623 (67.4%)
unknown	59 (15.1%)	93 (13.6%)	150 (16.3%)

*: p < 0.05, compared to the respective group of subjects without chronic diseases

**: p < 0.01, compared to the respective group of subjects without chronic diseases).

## Discussion

Our South-East Hungarian population-based study is the first to establish the epidemiologic characteristics of GERD-related symptoms in Eastern Europe. Our results showed a significantly lower prevalence of these symptoms compared with those in Western countries. Although the studied population was likely healthier than the general population, GERD-related symptoms were detected and associated with different socioeconomic and other risk factors, such as positive family history, obesity, coffee consumption, and smoking.

Most population-based epidemiological studies have reported a high prevalence of GERD-related typical symptoms appearing at least monthly. In general, prevalence ranges from 20% to 30% in Western countries. A study in the UK, which enrolled 3,179 patients, reported GERD in 28.7% of the sample population and found it was more common among socially disadvantaged individuals (*P* < 0.005). Although we also examined these potential associations, we were unable to show a correlation between the presence of GERD-related symptoms and the different measurements of socioeconomical status of our participants. Another study conducted in the USA reported that the prevalence of heartburn and/or acid regurgitation experienced at least weekly was 19.8%. In that study, heartburn and acid regurgitation were associated with noncardiac chest pain, dysphagia, dyspepsia, and globus sensation but not with asthma, hoarseness, bronchitis, or history of pneumonia. A recent Italian study reported that the prevalence of gastro–esophageal reflux was 26.2% (792/3012). The authors found significant differences in frequency of the disorder according to sex, smoking habits, and BMI. GERD-related symptoms were more common among females, smokers, and those with higher BMI values [18–20].

In contrast, a study conducted in India reported a lower prevalence of GERD-related symptoms, with 7.6% of the 3,224 participants experiencing heartburn and/or acid regurgitation at least once a week. Older age and consumption of non-vegetarian, fried foods, aerated drinks, and tea/coffee were associated with GERD. Frequency of smoking and BMI were similar among participants with or without GERD [21].

A study conducted in Iran including 803 patients (age, 11–84 years) reported that GERD was more common in females than males. Furthermore, the disease became more prevalent with age. In the present study, there was an interesting association between sex and the presence of GERD-related symptoms. As the frequency of GERD-related symptoms increased, the blood donors were more likely to be female. In our participants, there was no difference according to age [22].

We identified only one study in the literature that was conducted in Japan that reported a positive relationship between the upper gastrointestinal symptoms and shorter height (in elderly, mostly female Japanese participants). In our blood donor volunteers, this correlation was also detected, and the height became shorter as the frequency of GERD-related symptoms increased [23].

Obesity plays a role in the development of GERD symptoms as well as its complications (erosive esophagitis, Barrett’s esophagus, and esophageal adenocarcinoma) [24]. Obesity was a detected risk factor in this population but the presence of overweight or obesity alone was not associated with a higher prevalence of symptoms. Most recent epidemiological studies detected an association between BMI and GERD, for both the symptomatic form and various complications (e.g., erosive esophagitis, Barrett’s esophagus). It should be also highlighted that obesity is one of the major risk factors of obstructive sleep apnea (OSA). The international literature demonstrates a high incidence of LPR (45.2%) in OSA patients. Moreover, a recent meta-analysis showed a significant correlation between OSA-hypopnea syndrome and GERD [25, 26]. Therefore, obesity has become a risk factor for these diseases. The present study examined this feature using two approaches. First, participants with mild, chronic, non-exclusionary diseases showed a greater frequency and prevalence of GERD-related (typical/atypical) symptoms than overweight/obese, otherwise healthy participants. Second, a positive linear correlation was found between the prevalence of GERD-related symptoms and the presence of obesity. In another study, the prevalence, frequency, and severity of symptoms of GERD increased with increasing BMI [27].

Smoking significantly exacerbates GERD via direct provocation of acidic reflux and a long-lasting reduction of lower esophageal sphincter pressure [28]. In our study, smoking was also a significant risk factor (Tables 3 and 4).

It remains unclear whether coffee consumption is a factor in the development of GERD. A recent study reported no association between coffee consumption and the symptoms or erosive esophagitis [29]. In another study, coffee (in contrast to tea) increased the prevalence of GERD. Therefore, factors other than caffeine may be responsible for the induction of GERD [30]. Coffee consumption was a significant risk factor in our study population (Tables 3 and 4).

A meta-analysis of 26 cross-sectional studies and three case–control studies showed a potential association between drinking alcohol and risk of GERD. Increased alcohol consumption and frequency showed a stronger correlation with GERD [31]. This association was not observed in the present study.

Taken together, these findings indicate that the prevalence of GERD-related symptoms in South-East Hungary was closer to the Eastern values. Interestingly, some of the detected risk factors supported previous results from Eastern studies while others supported the findings from Western studies.

Some chronic diseases may be associated with upper gastrointestinal motility disorders. However, epidemiological studies of symptomatic GERD have not evaluated their influence on the prevalence of GERD-related symptoms. In the present study, an association was found between GERD-related symptoms and chronic diseases. Furthermore, their prevalence was three times higher in individuals with mild, chronic non-exclusionary diseases compared with apparently healthy individuals. Therefore, primary GERD is likely less common based on the findings from epidemiological studies. This difference may be explained by upper gastrointestinal hypomotility associated with these disorders or their pharmaceutical treatment.

Many risk factors are common in cardiovascular diseases and GERD and GERD can be a risk factor for cardiovascular diseases (such as hypertension). A significant correlation was previously found between GERD and hypertension, as well in our study population [32]. Our findings support some well-known symptom–disease associations, such as coughing and respiratory disorders, chest pain and heart diseases, and globus sensation and thyroid problems. All typical and atypical symptoms were significantly more common (*P* < 0.05) in individuals with known minor respiratory diseases (44/2,002, 2.2%).

Positive family history of GERD has been examined in several studies, but these mostly occurred for the genetic markers. Epidemiological studies have not examined the positive family history of the disease in larger populations along with GERD-related symptoms. Our findings indicate that individuals with a higher frequency of typical GERD symptoms were more likely have a positive family history of GERD. While the prevalence was twice as high for those experiencing less than weekly symptoms, the likelihood of a positive family history was three times higher in individuals with at least weekly symptoms [33, 34].

The present study has some limitations. The study was limited to a questionnaire survey of symptoms and medical history. No instrumental examinations (e.g., endoscopy and esophageal function testing, such as the gold standard pHmetry) were performed in the participants. Therefore, only the prevalence value of the examined symptoms could be given and there was no data to state whether symptoms were caused by GERD (acidic/weakly acidic/non-acidic reflux) or functional disease (e.g., functional heartburn). In this study, the lack of RSI score is a limitation, which is mainly used for LPR but can be also performed for GERD patients for an evaluation of the symptoms [35, 36]. Patients with OSA and / or obesity were not observed in our study because there were no questions for sleeping disorder in the questionnaire.

## Conclusion

In conclusion, the prevalence of GERD-related symptoms among South-East Hungarian blood donor volunteers was significantly lower than in the Western countries and closer to the Eastern values. In otherwise healthy, non-obese individuals, the prevalence of at least weekly occurring GERD-related symptoms was <5%. The presence of mild, non-exclusionary chronic diseases significantly increased the prevalence of GERD-related symptoms, as well as positive family history, coffee consumption, smoking, shorter height, and increased BMI.
